# Development of a Novel Carbon Emissions Estimation Tool for Disposable Waste Associated With Antimicrobial Packaging, Preparation, and Administration in the Hospital Setting

**DOI:** 10.1093/ofid/ofaf308

**Published:** 2025-10-10

**Authors:** Leila S Hojat, Courtney Veltri, Nicholas J Newman, Rachel E Ferreira, Victoria L Moore, Keli J Higbee-Todd, Alexis P Lee, Amanda G Woods, Mendel E Singer, Emily S Spivak

**Affiliations:** Division of Infectious Diseases, Department of Medicine, Case Western Reserve University, Cleveland, Ohio, USA; Division of Infectious Diseases, Department of Medicine, University Hospitals Cleveland Medical Center, Cleveland, Ohio, USA; Department of Pharmacy, University Hospitals Cleveland Medical Center, Cleveland, Ohio, USA; Department of Pharmacy, University Hospitals Cleveland Medical Center, Cleveland, Ohio, USA; Department of Pharmacy, University Hospitals Cleveland Medical Center, Cleveland, Ohio, USA; Department of Nursing, University Hospitals Cleveland Medical Center, Cleveland, Ohio, USA; Department of Nursing, University Hospitals Cleveland Medical Center, Cleveland, Ohio, USA; Environmental and Social Sustainability, University of Utah Health, Salt Lake City, Utah, USA; Pharmacy Services, University of Utah Health, Salt Lake City, Utah, USA; Department of Population and Quantitative Health Sciences, Case Western Reserve University, Cleveland, Ohio, USA; Division of Infectious Diseases, Department of Internal Medicine, University of Utah School of Medicine, Salt Lake City, Utah, USA

**Keywords:** antimicrobial stewardship, calculator, metrics, sustainability, waste reduction

## Abstract

**Background:**

Addressing climate change is one of the most pressing needs of society. The One Health approach recognizes the importance of antimicrobials at the intersection between humans, animals, and the environment and advocates for mitigation of antimicrobial overuse, primarily as a means of preventing antimicrobial resistance. Antimicrobial use additionally contributes to climate change through consumption of single-use disposable products used for packaging, drug preparation, and intravenous drug administration, which in turn generates greenhouse gas (GHG) emissions when disposed.

**Methods:**

We estimated the GHG emissions associated with intravenous antimicrobials given in the hospital setting, initially performing the data collection in a large academic hospital in Cleveland, Ohio, and validating the data in another large academic hospital in Salt Lake City, Utah. For each antimicrobial agent, we identified all disposable packaging, preparation, and administration materials. Materials were weighed and classified by type, and total GHG emissions measured in carbon dioxide equivalents were calculated using emission factors defined by the U.S. Environmental Protection Agency.

**Results:**

Results were summarized in tables listing GHG emissions per dose per antimicrobial agent, and a calculator tool was created in spreadsheet format to accommodate antimicrobial use data collected by hospital antimicrobial stewardship programs.

**Conclusions:**

We developed a novel tool for estimating GHG emissions associated with single-use waste generated from IV antimicrobial packaging, preparation, and administration in the hospital setting. This tool can be used by antimicrobial stewardship programs to assess their institutions’ GHG emissions and provide another stewardship value measure quantifying avoided GHG emissions with antimicrobial optimization strategies.

## Teaser

We developed a novel tool for estimating greenhouse gas emissions from single-use waste generated from IV antimicrobials in the hospital setting. This tool allows antimicrobial stewardship programs to assess their hospitals' antimicrobial-associated waste and measure the impact of stewardship interventions.

Climate change has become one of the leading threats to global public health. Extreme weather changes contribute to excess morbidity and mortality due to increases in food insecurity, heat and extreme weather–related deaths, and increased distribution of deadly infectious diseases [1, 2]. The health care sector is responsible for ∼4.4% of global net greenhouse gas (GHG) emissions, and the United States health care system accounts for 8.5% of domestic GHG emissions [3, 4]. Therefore, efforts to reduce GHG emissions within the health care sector are imperative in curbing the long-term health detriments secondary to climate change.

One Health is a collaborative effort that proposes an integrated approach to balance and optimize the connection between the health of people, animals, and ecosystems. Part of One Health includes examining the impact health systems have on the environment and identifying ways to reduce its contribution to climate change [5]. Antimicrobials are one of the most prescribed medications, and intravenous (IV) antimicrobials are associated with various single-use products that contribute to health care waste and global GHG emissions. When plastic, metal, cardboard, and other solid waste used in the delivery, preparation, and administration of drugs are landfilled, they release methane, carbon dioxide, and other greenhouse gases as they break down, and these gases in turn trap heat in the atmosphere [6, 7]. Moreover, >50% of hospitalized patients in the United States receive antimicrobials, and at least 30%–50% of these may be prescribed incorrectly or unnecessarily [8–10]. Interventions focused on early conversion of IV antimicrobials to oral therapy are often a key component of antimicrobial stewardship programs, and data indicate a cost benefit of such initiatives [11, 12]. However, there are no available data regarding the measurement of waste associated with each dose of an IV antimicrobial.

The objective of our study was to determine the GHG emissions due to single-use waste associated with individual antimicrobials given in the hospital setting. Further, we aimed to use these estimates to create an IV antimicrobial waste calculator that could be used to measure GHG emissions reductions for IV antimicrobials avoided. This calculator could then be used by antimicrobial stewardship programs and health care systems to estimate GHG emissions avoided through reductions in waste from unnecessary antimicrobial use.

## METHODS

This study was performed using waste materials collected throughout September 2024 from a large academic medical center in Cleveland, Ohio, as the primary institution with secondary confirmation of the results by a large academic medical center in Salt Lake City, Utah. The University Hospitals Institutional Review Board provided approval for this research under the protocol “Investigating the Environmental and Economic Impacts of Early IV to PO Antimicrobial Switch Therapy” (UH IRB STUDY20240291). This study was classified by the University of Utah Institutional Review Board as a quality improvement project and did not require review and oversight.

Our antimicrobial GHG emissions calculator was developed through the following steps: (1) cataloging and collecting all individual items used in antimicrobial packaging, preparation, and administration; (2) weighing each item; (3) designating a material type for each item; (4) calculating the GHG emissions for each item; and (5) totaling the GHG emissions used by all items required for a single dose and for a single day of therapy (DOT) for each agent. For validation purposes, infectious diseases pharmacy, pharmacy operations, and nursing at the secondary medical center reviewed the information collected from the primary center to confirm that the items, preparation processes, and administration practices were in alignment.

To initiate step 1, a preliminary list of IV antimicrobial agents to be included was generated based on the primary institution's formulary and standard adult dosing. Pharmacy inventory software and institutional IV medication guidelines review were used to classify antimicrobials as those requiring admixture compounding by an on-site pharmacy technician, powder to be reconstituted via the Mini-Bag Plus system (Baxter International Inc.), frozen premix, or nonfrozen premix. Each dosing strength and product type available for each drug were documented as separate drug products. Packaging for each item was collected, including paperboard boxes for powders, small corrugated boxes for premixes, and overwrap for premixes. Shipping materials were not included. Empty drug materials collected included discarded powder vials postreconstitution and premix bags postinfusion, ensuring no residual drug remained. Each item used in the pharmacy reconstitution process and all items used in drug administration were identified and collected, ensuring that items containing fluid (ie, sterile water, saline, dextrose in water) were empty and dry. Personal protective equipment used for sterile compounding or for drug administration requiring interaction with patients with isolation precautions were not included.

For step 2, each item was weighed using a tabletop scale accurate to the level of centigrams, and each item was classified to a material type according to the United States Environmental Protection Agency (EPA) Waste Reduction Model (WARM) to complete step 3. Items with multiple material components were categorized according to the most substantial component material. Items that could not definitively be classified into a specific material type were classified as a “mixed” type or according to the closest similar WARM material. Step 4 involved calculating waste-associated carbon dioxide (CO_2_)–equivalent GHG emissions based on the weight and type of each item using the EPA GHG emission factors listed in the EPA GHG Emission Factors Hub [13]. Different weight measurements collected between institutions were averaged. Waste emissions were calculated by multiplying the total mass of each item by the appropriate emission factor depending on the waste type (eg, glass, polypropylene, corrugated containers, etc.) and waste treatment method, which we designated as landfilled, the method of disposal used for both institutions’ nonhazardous waste. Recognizing that some institutions may recycle or incinerate certain materials, we provided the ability to modify the method of disposal, allowing institutions to determine their own estimated GHG emissions more accurately.

To fulfill the final step in developing the emissions tool, a pharmacy operations coordinator from each site (R.F., A.W.) determined the number of each item used in the preparation of each antimicrobial dose. Likewise, 2 nurse managers from the primary institution (V.M. and K.H.) and nursing staff at the secondary institution assisted with establishing the average count of each administrative item used for infusing antimicrobials. All packaging, preparation materials, and administration supplies were tallied, and the corresponding emissions determined in step 4 were applied in order to derive the total admissions for a single dose of each drug product. To account for differences in preparation and administration between sites, the total CO_2_-equivalent GHG emissions for each drug product were calculated based on data from each site and subsequently averaged. To determine the emissions per DOT per drug, the total emissions from packaging and preparation were multiplied by the number of doses of the drug typically given per day, assuming normal renal function and standard dosing for the most common indication. The total emissions per day per drug were added to the calculated emissions from administration materials used each day to arrive at a final estimated CO_2_-equivalent GHG emissions per DOT per agent. For antimicrobials with multiple doses, GHG emissions data were averaged within categories of agents (for the DOT Calculator) or categories of agents and doses (for the dose calculator).

## RESULTS

**Figure 1.**
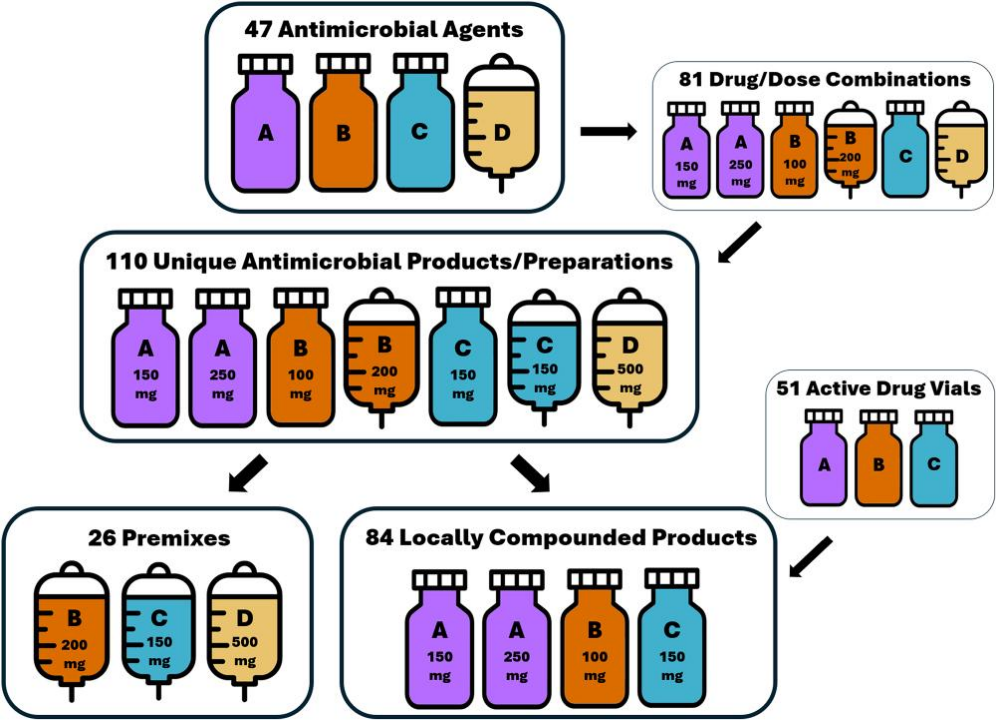
Diagram of antimicrobial drug products included in the study. Vials signify products requiring reconstitution from powder (ie, admixture requiring local compounding or Mini-Bag Plus system), while bags signify premix products (ie, frozen or nonfrozen premixes).

We identified 110 unique admixtures, which consisted of 26 premixes and 84 locally compounded products, representing 47 antimicrobial agents with 81 unique agent/dosing strength combinations (Figure 1). Certain active drug vials were used for multiple dosing strengths of the same antimicrobial agent (eg, acyclovir 500-mg vial used for both acyclovir 250 mg/50 mL and acyclovir 500 mg/100 mL products) or in different volumes. Locally compounded products included 51 different active drug vials, of which 23 (45%) were measured by both institutions. Seventeen (74%) had a <15% difference between weights, and 4 (17%) had a >25% difference. The agents, formulation types, weights, WARM material classifications, and calculated GHG emissions for each drug product and drug-specific packaging materials are included in Supplementary Table 1. Additionally, we identified 102 separate inactive materials used for antimicrobial preparation or administration, with the corresponding information included in Supplementary Table 2. Of these, 46 materials (45%) were measured at both sites, 28 of which (61%) had a <15% difference and 13 (28%) of which had a >25% difference. The total calculated GHG emissions generated from daily administration materials were found to differ between institutions by ∼37%. The tallied counts of materials used for preparation of each agent are listed in Supplementary Table 3. Premix box and drug vial paperboard box counts were divided by the number of each item included per box (eg, 12 cefazolin 1 g/50 mL frozen premix items included per box = 0.08 boxes per dose). Preparation materials were divided by the number of doses derived if multiple doses were from each preparation process (eg, 3–3 g/100 mL doses derived from each cefazolin 10 g vial = 0.33 vials per dose). Total number of doses per day for each drug product are also included in Supplementary Table 3. Total CO_2_-equivalent GHG emissions for each drug product were calculated and listed in Supplementary Table 4, and total drug masses for each product are listed in Supplementary Table 5; each of these tables references the drug and material weights and emissions from Supplementary Table 1 and Supplementary Table 2, then multiplies by the counts in Supplementary Table 3 and averages the values generated from the institutions. The final summary and averages of drug product CO_2_-equivalent GHG emissions are listed in Supplementary Table 6. Of the 110 drug products, 26 had identical volumes and formulations at both institutions, with total emissions related to preparation differing by 20%. The reference table for weights and emissions summarized from the EPA GHG Emission Factors used to calculate the emissions is included in Supplementary Table 7.

**Figure 2.**
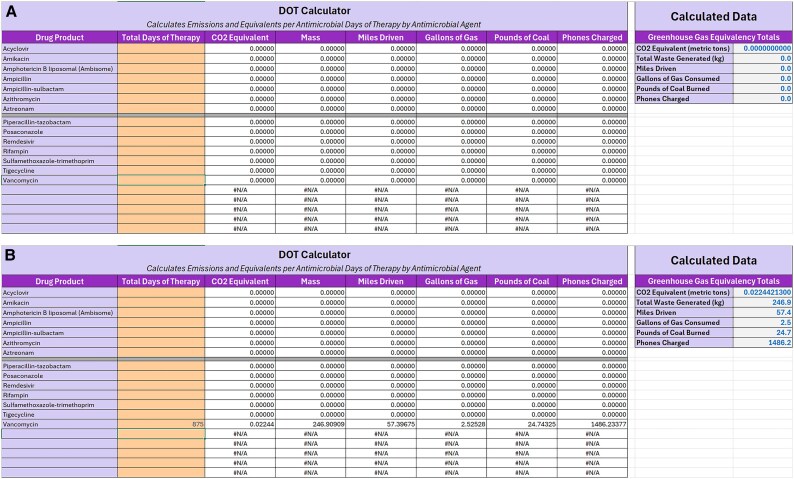
Screenshots of days of therapy (DOT) calculator. A, Calculator before data entry. B, Calculations automatically applied after entering data for vancomycin.

The Antimicrobial Emissions Calculators were created using the final calculated CO_2_-equivalent GHG emissions values, which are listed in the “Reference” tab along with the emission factors for the GHG equivalents used in the 2 calculator tabs. The “DOT Calculator” includes a simplified list of agents compatible with information compiled from standard antimicrobial use data such as data compiled from the Centers for Disease Control and Prevention's National Health care Safety Network (NHSN) line listing report. The number of IV DOT are input in column B next to the corresponding agent (Figure 2). Column C calculates the CO2 equivalent in short tons, Column D calculates the total mass of the waste generated, and Columns E through H calculate various equivalencies for interpretation. The calculated data are then summed along each column, with the totals listed in Column K in rows 3 through 8. The “Dose Calculator” and “Drug Product Calculator” work analogously but can accommodate more granular data if these are available.

## DISCUSSION

The findings from this study provide a novel metric for assessing the value of antimicrobial stewardship programs through prevented GHG emissions from avoided IV antimicrobials. Previous stewardship studies have predominantly reported on outcomes such as drug cost, length of stay, and adverse effects; however, sustainability and environmental impacts are crucial measures and may provide a more motivating outcome to influence prescribers [14, 15]. The nontraditional measurements in this study support the One Health approach by examining human health care impact on the environment. The environment, by way of climate change, affects human health by providing factors optimal for the spread of health care–associated infections and antimicrobial resistance proliferation [5, 16]. As prevention of these consequences is one of the primary goals of antimicrobial stewardship programs, incorporation of environmental impact outcomes in programs is essential, and the impact is expected to be substantial, with >50% of inpatient antimicrobials administered estimated to be unnecessary [17]. These measures also have implications for daily stewardship practice, with sustainability factors like dosing frequency and drug formulation further complicating the antimicrobial optimization process when considered alongside traditional elements such as spectrum and route [18].

Contributors to climate change are often described from a global perspective, and the ability to conduct a local assessment and evaluation of impacts to climate change can be challenging. In detailing the process of our assessment, we hope to encourage others to conduct studies of potential waste in their health care environments. Notably, this is one of the first publications to provide a tool for direct estimation of GHG emissions related to antimicrobial use and, to our knowledge, the first to estimate waste from inpatient IV antimicrobials. IV-to-oral (PO) conversion is a common initiative employed by stewardship programs, often with the assistance of pharmacy staff; however, adherence to these policies may not be optimal [19, 20]. Having a quantifiable process of determining the environmental impact of often unnecessary IV antimicrobial use may influence programs to strengthen these efforts. To illustrate how this may be integrated into existing programs, the example data provided in Figure 2 demonstrate a calculation of the estimated avoided GHG emissions based on a publication describing a decrease of ∼875 DOT of vancomycin resulting from a pharmacy-initiated MRSA nasal polymerase chain reaction (PCR) test intervention, equivalent to GHG emissions generated from 57 miles driven [21]. These initiatives apply to nonantimicrobial pharmaceuticals as well, given that other classes of medications with highly bioavailable oral formulations, such as proton pump inhibitors and anticonvulsants, are likewise common targets of IV-to-PO interventions [22, 23]. Finally, similar methods could be used to examine and quantify at a local level the myriad additional factors contributing to the carbon footprint of health care processes beyond the topic of IV-to-PO conversion [24, 25].

This study includes several limitations. First, the quantified CO_2_-equivalent GHG emissions only include the materials being disposed of by the hospital, but these are underestimates, not accounting for the additional major sources of antimicrobial-associated GHG emissions such as the drug manufacturing process and shipping [26]. Lack of transparency in the drug manufacturing process limits the extent to which information can be collected and quantified at the same granular level used for our materials assessment. Moreover, shipping-related GHG emissions are variable depending on location and shipping quantity. Other sources of GHG emissions not accounted for by our calculator include personal protective equipment, refrigeration, and GHG emissions related to antimicrobial-associated complications. Second, minor variations in measurements between our 2 hospitals likely exist due to drug products from different manufacturers, and our hospitals may not carry or have available the same formulations of medications as other hospitals. For example, we may use a premix antimicrobial where others may use an admixture for the same agent. Institutions may also carry different antimicrobials on their formulary. Ideally, manufacturers would provide information on single-use waste-associated GHG emissions from their products, so that health care systems could factor sustainability into purchasing decisions. Also, supply chain shortages may affect an institution's product utilization or compounding practices. Given these anticipated differences, we provided the complete list of agents and the calculations used to create our final calculator in our Supplementary Data so that these could be adapted if needed by other institutions. Similarly, the calculator could be adjusted for differences in the process or products used for reconstituting drugs. Another limitation of the study relates to our estimation of materials used in antimicrobial administration, as practices vary even within the same institution based on factors related to the specific patient as well as personal practice. For example, the ability to maintain tubing for an individual patient is affected by whether they have been connected continuously to fluids and if nursing has accurate documentation of when the tubing was first setup. Using a secondary infusion line also may not be the standard practice at every institution. We based our calculations on the anticipated average materials used per day, but the calculator also may require adjustment for these differences to achieve the greatest accuracy for individual hospitals. Lastly, our DOT Calculator may provide overestimates for patients with impaired renal function receiving fewer doses, though we anticipate this to be negligible as this does not apply to all agents and is likely overcompensated for by the underestimates described. Given that DOT is typically calculated from administered doses, another potential underestimate could result from doses being prepared without being administered.

Our study also has notable strengths. Despite the potential for individual variation, our calculator is expected to provide a meaningful estimate for the average materials used in antimicrobial preparation and administration generalizable to most institutions based on our involvement of a multidisciplinary team comprising infectious diseases physicians, infectious diseases pharmacists, nursing staff, nurse managers, and pharmacy operations coordinators across 2 large academic centers located in distinct US regions. We confirmed approximately half of our data at both sites and noted ∼60%–75% concordance between product weights and materials, as well as ∼80% agreement on drug preparation. Another strength of our study is the simple and convenient format of our calculator, which has been designed specifically to accommodate data that most stewardship programs have access to. DOT data are readily attainable from pharmacy systems and in the US from NHSN data, which is now a mandatory component of the Promoting Interoperability Program. Alternatively, more precise estimation may be determined using the alternative calculators if these data are available, which may help account for additional variations such as renal impairment as mentioned above, loading doses, single doses used for prophylaxis, and Defined Daily Dose (DDD) data. Finally, we have provided all information used to derive the calculators within the Supplementary Data to allow for institution-specific adjustments if individual programs have more specific or additional data to be included or different disposal methods to account for, as well as partially populated fields to allow for new data integration.

This study provides the foundation for several potential future projects. It would be worthwhile to demonstrate the utility of the calculator through application to real-world data, including baseline antimicrobial use among specific hospitals or hospital systems, quantifying the impact of antimicrobial stewardship interventions, or estimation of GHG emissions related to antimicrobial overuse at a higher level analogous to recent work evaluating GHG emissions related to ambulatory prescriptions [27]. Additionally, the calculator tool may be expanded to other populations or medications, such as pediatric antimicrobial dosing or nonantimicrobial intravenous medications. From the perspective of evaluating the impact of IV-to-PO conversion programs, calculating CO_2_-equivalent GHG emissions related to oral agents would also be useful. Oral antimicrobials are anticipated to have significantly lower but still quantifiable associated waste primarily secondary to packaging and distribution in the hospital setting. For example, some hospitals distribute each drug in separate plastic bags or envelopes, and medications may be placed into paper or plastic cups for easier patient consumption.

In addition to estimating GHG emissions, there are many other measurable savings related to transitioning from IV to PO agents, including personnel time, length of stay, and complications related to IV access, that could be included as part of a cost savings model [28, 29]. In performing our study, we observed pharmacy technicians spending between ∼4 and 13 minutes preparing and verifying individual IV antimicrobial doses, depending on product type. Nursing time to obtain and administer each IV antimicrobial dose generally ranged between 3 and 17 minutes, depending on patient IV access, isolation precautions, existing infusion setup, and other medications concomitantly administered. Considering that each patient may be receiving several doses of several different IV antimicrobials per day, the potential cost savings of nursing and pharmacy technician time by transitioning IV antimicrobials to PO are likely considerable and should be evaluated systematically. Finally, future studies should continue to develop tools and metrics to estimate the carbon footprint of other waste components of health care in order to promote transparency and accountability, set standards, and measure the impact of interventions to reduce health care–associated GHG emissions.

## CONCLUSIONS

An important component of the health care carbon footprint includes utilization of intravenous agents. We developed a novel tool for calculation of the estimated GHG emissions associated with single-use waste generated from IV antimicrobial packaging, preparation, and administration in the hospital setting. Antimicrobial stewardship programs can utilize this tool to measure their baseline IV antimicrobial–associated GHG emissions and the relative impact of interventions to reduce overall antimicrobial use or to transition IV agents to PO.

## Supplementary Material

ofaf308_Supplementary_Data

## Supplementary Data

Supplementary materials are available at *Open Forum Infectious Diseases* online. Consisting of data provided by the authors to benefit the reader, the posted materials are not copyedited and are the sole responsibility of the authors, so questions or comments should be addressed to the corresponding author.
