# Supplementary material for: Development of a Novel Carbon Emissions Estimation Tool for Disposable Waste Associated With Antimicrobial Packaging, Preparation, and Administration in the Hospital Setting
Source: Open Forum Infect Dis. 2025 Oct 10;12(10):ofaf308. doi: 10.1093/ofid/ofaf308 (PMC12548793; doi:10.1093/ofid/ofaf308)
Supplement: ofaf308_Supplementary_Data [file ofaf308_supplementary_data.zip › Supplementary_Materials.docx]

**Supplementary Materials:**

**Antimicrobial Emissions Calculator (xlsx file)**

- **DOT Calculator:** Calculates Carbon Dioxide Equivalents per Antimicrobial Days of Therapy by Antimicrobial Agent.
- **Dose Calculator:** Calculates Carbon Dioxide Equivalents per Antimicrobial Dose by Antimicrobial Agent and Dose.
- **Drug Product Calculator:** Calculates Carbon Dioxide Equivalents per Antimicrobial Dose by Antimicrobial Agent, Dose, and Formulation.
- **Reference:** Summary of reference values used for Antimicrobial Emissions Calculators.

**Supplementary Tables (xlsx file)**

- **Table S1**: **Drug Products**
  Full list of antimicrobial drug products, vial type (for admixtures), vial and packaging weights, and calculated carbon dioxide-equivalent greenhouse gas emissions based on material and waste disposal method.
- **Table S2: Preparation and Administration Materials**
  Full list of pharmacy antimicrobial preparation and antimicrobial infusion administration materials, item weights, and calculated carbon dioxide-equivalent greenhouse gas emissions based on material and waste disposal method.
- **Table S3: Drug Preparation Material Counts**
  Tally of materials used in the preparation of each drug product and total doses designated per drug product per day.
- **Table S4: Drug Product Emission Calculations**
  Calculates final total metric ton carbon dioxide equivalents for each drug product by combining emissions, tallied material counts, and dosing frequency data from Tables S1, S2, and S3.
- **Table S5: Drug Product Mass Calculations**Calculates final total mass in kilograms for each drug product by combining drug-specific weights, tallied material counts, and dosing frequency data from Tables S1, S2, and S3.
- **Table S6: Emissions Data References for Calculators**
  Summary of emissions data calculated in Table S4 serving as reference values for the Antimicrobial Emissions Calculators.
- **Table S7: Reference Material**Greenhouse gas emissions data reference material and unit conversions.
